# P-1592. Evaluating the Effectiveness of 2024-2025 Seasonal mRNA-1273 Vaccination Against COVID-19-Associated Hospitalizations and Medically Attended COVID-19 among adults aged ≥ 18 years in the United States

**DOI:** 10.1093/ofid/ofaf695.1771

**Published:** 2026-01-11

**Authors:** Amanda Wilson, Alina Bogdanov, Gigi Zheng, Taylor Ryan, Ni Zeng, Keya Joshi, Tianyi Lu, Machaon Bonafede, Andre B Araujo

**Affiliations:** Moderna, Inc., Cambridge, Massachusetts; Veradigm, Chicago, Illinois; Moderna, Inc., Cambridge, Massachusetts; Veradigm, Chicago, Illinois; Veradigm, Chicago, Illinois; Moderna, Inc, Cambridge, Massachusetts; Moderna, Inc., Cambridge, Massachusetts; Veradigm, Chicago, Illinois; Moderna, Inc., Cambridge, Massachusetts

## Abstract

**Background:**

This study evaluated the effectiveness of Moderna’s updated mRNA-1273 vaccine targeting the KP.2 variant, compared to people who did not receive any 2024-2025 COVID-19 vaccine, in preventing COVID-19-associated hospitalizations and medically attended COVID-19 among adults aged ≥18 years in the United States during the 2024-2025 season.

**Figure:**
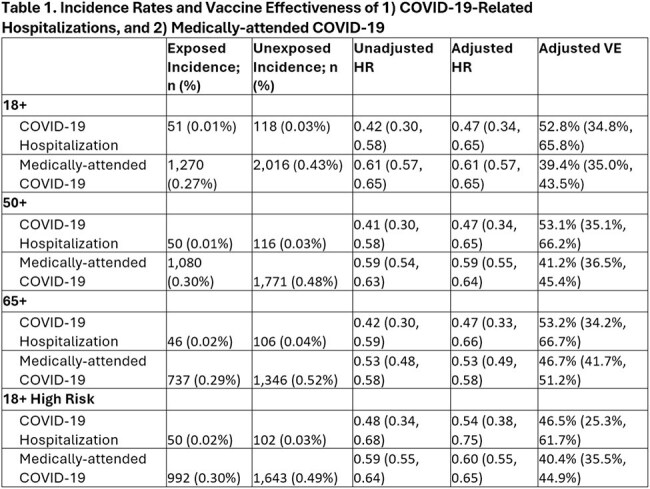

**Methods:**

Data were extracted from linked administrative healthcare claims and electronic health records (EHR) for vaccinations from 23 August 2024 through 24 December 2024 and followed through 31 December 2024. We conducted a retrospective matched cohort study with propensity score weighting to adjust for differences between groups to assess vaccine effectiveness (VE) against COVID-19 outcomes. VE was calculated as 1 minus the hazard ratio (HR) from Cox proportional hazards models.

**Results:**

Overall, 465,073 mRNA-1273 KP.2 vaccine recipients were matched 1:1 to unexposed adults. The mean (standard deviation) age was 63 (17) years, with more than half of the population being 65 years or older. Approximately 70% of individuals had an underlying medical condition making them high risk for severe outcomes for COVID-19. VE was 52.8% (95% confidence interval [CI], 34.8%, 65.8%) against COVID-19–related hospitalization and 39.4% (35.0%, 43.5%) against medically attended COVID-19 over a median follow-up of 57 (interquartile range: 33-78) days (Table 1).

**Conclusion:**

The mRNA-1273 KP.2 vaccine demonstrated significant incremental effectiveness in preventing COVID-19–related hospitalizations and medically attended COVID-19 in adults during the 2024-2025 season to date. These findings support ongoing vaccination efforts to mitigate the public health impact of COVID-19.

**Disclosures:**

Amanda Wilson, PhD, Moderna, Inc.: Employee|Moderna, Inc.: Stocks/Bonds (Public Company) Alina Bogdanov, MA, Veradigm: Employee Gigi Zheng, MD, PhD, ModernaTX: Employee|ModernaTX: Stocks/Bonds (Public Company) Taylor Ryan, MHI, Veradigm: Employee Ni Zeng, PhD, Veradigm: Employee Keya Joshi, PhD, Moderna, Inc.: Employee|Moderna, Inc.: Stocks/Bonds (Public Company) Tianyi Lu, PhD, Moderna, Inc.: Employee|Moderna, Inc.: Stocks/Bonds (Public Company) Machaon Bonafede, PhD, Veradigm: Employee Andre B. Araujo, PhD, Moderna, Inc.: Employee|Moderna, Inc.: Stocks/Bonds (Public Company)

